# Quantitative real time PCR for distinction between *Pneumocystis jirovecii* infection/colonization in hospitalized patients

**DOI:** 10.3389/fcimb.2024.1426200

**Published:** 2024-09-24

**Authors:** Faezeh Rouhi, Mahzad Erami, Sepide Rastgufar, Maryam Jahani, Shima Aboutalebian, Sajedeh Soltani, Hamed Fakhim, Hossein Mirhendi

**Affiliations:** ^1^ Department of Medical Parasitology and Mycology, School of Medicine, Isfahan University of Medical Sciences, Isfahan, Iran; ^2^ Department of Infectious Disease, School of Medicine, Infectious Diseases Research Center, Kashan University of Medical Sciences, Kashan, Iran; ^3^ Department of Pathology and Histology, School of Medicine, Shahid Beheshti Hospital, Kashan University of Medical Sciences, Kashan, Iran; ^4^ Mycology Reference Laboratory, Research Core Facilities Laboratory, Isfahan University of Medical Sciences, Isfahan, Iran; ^5^ Infectious Diseases and Tropical Medicine Research Center, Isfahan University of Medical Sciences, Isfahan, Iran

**Keywords:** *Pneumocystis jirovecii*, diagnosis, qPCR, nested PCR, Iran

## Abstract

**Background:**

Identification of the opportunistic fungus *Pneumocystis jirovecii* in respiratory specimens presents challenges, particularly in differentiating between colonization and active infection. The present study assessed a probe-based real time PCR (qPCR) diagnostic effectiveness in patients with diverse underlying conditions, particularly those with COVID-19 and pulmonary insufficiency.

**Methods:**

To set up the qPCR, clinical samples from 281 patients with respiratory ailments were tested. Subsequently, a descriptive study was conducted on 112 patients with pulmonary insufficiency with and without COVID-19 suspected of *P. jirovecii* infection. All specimens were subjected to DNA extraction followed by nested PCR and qPCR targeting the mitochondrial large subunit (mtLSU)-rRNA gene.

**Results:**

Based on nested PCR and qPCR, *P. jirovecii* was identified in 40 out of 281 patients, with slight variations in positive samples observed across dilutions. Three patients who tested positive in nested PCR yielded negative results in probe-based qPCR. Conversely, three patients who tested positive in probe-based qPCR yielded negative results in nested PCR. Considering nested PCR as the golden standard, probe-based qPCR demonstrated good diagnostic performance, with 92.5% sensitivity and 98.7% specificity. Based on cycle threshold (Ct) values, the positive cases were categorized: ≤32 as infection, >35 as colonization, and a grey zone between these values (32 < X ≤ 35). The analysis of 112 PCP-suspected patients revealed a prevalence ranging from 6.25% (nested PCR) to 7% (probe-based qPCR).

**Conclusions:**

This study suggested Ct values to differentiate *Pneumocystis* pneumonia/colonization in immunocompromised patients. To further augment the diagnostic sensitivity, it is recommended to integrate qPCR results with clinical parameters and biomarkers to offer a more precise understanding of *Pneumocystis*-related conditions.

## Introduction

*Pneumocystis jirovecii* (previously known as *P. carinii*) is the fungus that causes *Pneumocystis* pneumonia (PCP), an acute and life-threatening lung disease (Alshahrani et al., 2020). This disease primarily affects immunocompromised patients who have conditions like organ transplants, HIV infection, hematologic malignancies, solid cancers, and individuals receiving immunosuppressive treatment, chemotherapy, or biotherapy due to T-cell deficiency (Fauchier et al., 2016; Yang et al., 2020). Recently, COVID-19 patients receiving immunomodulatory therapies like corticosteroids experienced enhanced *P. jirovecii* colonization/infection, increasing the risk of co-infection and disease severity (Razazi et al., 2021). In addition, it is commonly reported in immunocompetent individuals (Linssen et al., 2006). Prompt and reliable diagnosis is imperative, especially in HIV-negative patients, because untreated PCP is associated with high morbidity and mortality.

Several factors can complicate the diagnosis of PCP, including nonspecific symptoms, concurrent infections, and lack of a cultivation system for this pathogen (Alshahrani et al., 2020). As *P. jirovecii* cannot be cultured/grown *in vitro*, diagnosing traditionally depends on direct microscopic examination of respiratory specimens (Yang et al., 2020). Significant drawbacks of microscopy include its cumbersome nature, time-consuming process, the requirement for a large specimen volume for optimal concentration, and the need for trained microscopists for reliable observation (Albulushi et al., 2021). Therefore, nucleic acid amplification tests, most commonly PCR, have been used to diagnose PCP (White et al., 2019). Nested PCR targeting the *Pneumocystis* mitochondrial large subunit (mtLSU)-rRNA gene or the multicopy major surface glycoprotein (msg) gene remains the most extensively used technique to identify *P. jirovecii* (Ma et al., 2018). However, a positive nested PCR result is unable to differentiate infection from colonization (Yang et al., 2020). Instead, real time PCR (qPCR) is a specific, sensitive, and quantitative technique that has the potential to differentiate between asymptomatic carriers of *P. jirovecii* and colonization from clinical infection based on the copy number of target genes, utilizing various cutoff values and enabling prompt initiation of appropriate therapy (Linssen et al., 2006; Alshahrani et al., 2020). Current guidelines recommend using qPCR to identify PCP (Donnelly et al., 2020).

The present study investigated *Pneumocystis* colonization and pneumonia in immunocompromised patients based on qPCR cycle threshold ​​(Ct) values. After establishing the method, the epidemiological aspects of *P. jirovecii* were studied in patients with respiratory insufficiencies with or without COVID-19 infection, who referred to a referral hospital. Our study offers new insights by addressing both methodological and epidemiological aspects of *P. jirovecii* detection. In addition, we compared the sensitivity and specificity of real time PCR versus nested PCR, providing a comprehensive analysis of the effectiveness of these diagnostic methods in detecting *P. jirovecii*. This comparison is crucial for improving diagnostic accuracy and treatment outcomes in immunocompromised patients.

## Materials and methods

### Samples collection

This descriptive study was conducted over two years, from March 2021 to August 2023, involving patients with pulmonary insufficiency referred to the Shahid-Beheshti referral hospital in Kashan, Iran. Patients with or without COVID-19 who had pulmonary symptoms and presented acute respiratory infections suspected of PCP were included in the study. **Table 1** displays the patients’ clinical characteristics. A total of 112 samples, including 36 bronchoalveolar lavage (BAL) fluids, 69 tracheal aspiration (TA), and seven sputa, obtained from 112 patients, were collected and stored at -20˚C until use. The samples were centrifuged at 5000 rpm for 10 min, and 400 µl of the sediment was transferred into a 1.5 mL tube for analysis. To liquefy the viscous sputum samples, an equal volume of 0.5% Pancreatin was added and incubated at 37˚C for 1 hour, followed by centrifugation. This study was authorized by the ethical committee of Isfahan University of Medical Sciences (IR.ARI.MUI.REC.1401.170). In addition, a set of 281 samples from patients with respiratory diseases, including 128 TA samples, 96 BAL, and 57 sputum, were used to establish/set up the qPCR. Epidemiological findings from these 281 samples have already been published (Matouri et al., 2023).

**Table 1:** Characteristics of tested patients with or without PCP.

Characteristics	All patients (n =112)	PCP patients (n =8)
Age (years), median (range)	59.7 (0–93)	50.25 (30–82)
Sex, male/female	71/41	7/1
Underlying diseases COVID-19 COPD Asthma Lung nodule and abscess Pneumonia Diabetes mellitus Heart diseases Trauma Surgery Others	32 9 8 11 20 21 12 10 8 13	3 1 2 2 1 2 - 1 2 2
Cancer Lung cancer Lymphoma Acute lymphoblastic leukemia Others	13 7 1 1 4	1 1 - - -

### Nested PCR

DNA was manually extracted using the previously described phenol-chloroform method (Matouri et al., 2023), and the purified DNA was stored at -20°C until PCR amplification. As there is no internationally accepted protocol to classify PCP based on clinical probability, we utilized nested PCR targeting mtLSU-rRNA gene (Matouri et al., 2023) as a sensitive method for molecular detection of *P. jirovecii*. In the first round of the PCR, the primer set pAZ102-H (5´-GATGGCTGTTTCCAAGCCCA-3’) and pAZ102-E (5´-GTGTACGTTGCAAAGTACTC-3’) amplifying a 346 base pair (bp); and in the second round the primers pAZ102-X (5´-GTGAAATACAAATCGGACTAGG-3’) and pAZ102-Y (5´-TCACTTAATATTAATTGGGGAGC-3’) amplifying a 267 bp fragment were used as already described (Matouri et al., 2023). DNA samples underwent the nested PCR in three dilutions of 1, 1/10, and 1/50. Stringent contamination prevention measures were employed throughout the process. A non-template control (distilled water instead of DNA) to monitor contamination and a DNA sample already proven to have PCP as the positive control were included in each PCR run. An aliquot of 5 μl of each nested PCR product was electrophoresed on 1.5% agarose gel containing 0.5 μg/ml ethidium bromide and observed under ultraviolet light.

### qPCR

*P. jirovecii*-specific primer set (PjF: 5’-CTGTTTCCCTTTCGACTATCTACCTT-3’ and PjR: 5´-CACTGAATATCTCGAGGGAGTATGAA-3’), and TaqMan probe (PjSL: 5´-6-fam-TCGCACATAGTCTGATTAT-BHQ-1-3’) targeting a 121 bp region of the mtLSU-rRNA gene (Alanio et al., 2011) were used for qPCR assay performed by Roche LightCycler 96 machine (Roche Molecular Biochemicals, Mannheim, Germany). The PCR assay was performed with a final volume of 20 µl containing 10 µl of the 2x master probe-mix (PCR biosystems, London, UK), 0.25 µM of each primer, 0.15 µM of the probe, and 2.5 µl of 1/10 dilutions of DNA samples. After pre-incubation of 2 min at 95˚C, the amplification consisted of 45 cycles of 15s 95˚C and 45s 56˚C. Furthermore, the mtLSU-rRNA PCR product was cloned to prepare positive control and the plasmid template to establish the qualitative probe-based qPCR. Briefly, a DNA fragment product amplified with the mtLSU-rRNA primers (PjF and PjR) was cloned into the pUCM-T vector following the manufacturer’s instructions (BIO BASIC, UK). The plasmid was propagated in *Escherichia coli* strain TOP10 cells. Positive colonies were cultured in LB-amp broth at 37°C for 24 hours and then purifying plasmid DNA with the FavorPrep Plasmid DNA Extraction Mini kit (Favorgen, Austria). DNA concentration, quantity, and purity were assessed at 260 nm wavelength using a Biowave II system. Tenfold serial dilutions (10^-5^ to 10^-11^) of the plasmid were prepared for sensitivity evaluation and standard curve establishment.

### Sequencing

To confirm the specificity of the qPCR, randomly selected positive qPCR products were purified and subjected to Sanger sequencing (Core Facilities Laboratory, Isfahan, Iran) with the primer PjF, using BigDye™ Terminator v3.1 Cycle Sequencing Kit (Applied Biosystems, USA), and the sequences were subjected to BLAST analysis (https://blast.ncbi.nlm.nih.gov/Blast.cgi).

### Statistical analysis

Statistical analyses were performed using the SPSS software version 22. Compared with the nested PCR results, qPCR results were evaluated by calculating the specificity, sensitivity, negative predictive values (NPV), and positive predictive values (PPV). The chi-square test was employed to evaluate the relationships between the categorical variables. P-values below 0.05 (<0.05) were regarded as significant.

## Results

### Setting up results

**Figure 1:**
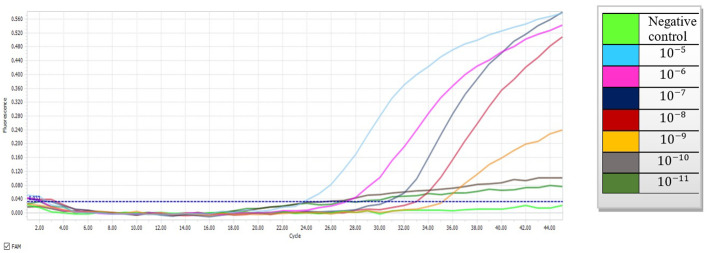
Amplification curves depicting tenfold serial dilutions of plasmid DNA containing the mtLSU-rRNA gene using probe-based qPCR (10^-5^ to 10^-11^).

To determine the performance of the probe-based qPCR assay and differentiation of colonization from infection, tenfold serial dilutions of the plasmid carrying the mtLSU-rRNA target gene (10^-5^ to 10^-11^) were prepared, and a standard graph (Ct versus log concentration) obtained from the amplification of the target gene in serial dilutions (10^5^– 1 copies) of the plasmid was constructed (**Figure 1**). In the qPCR, the detection limit was less than 10 copies of the plasmid per reaction. The repeatability of the assay was demonstrated by testing the serial dilutions in duplicates, and all positive control dilutions demonstrated success with the settings, indicating that it is suitable to proceed with clinical specimens. (**Table 2**).

**Table 2:** The sensitivity of probe-based qPCR for the detection of *P. jirovecii* DNA.

Sample Dilution	Cloned 121-bp *P. jirovecii* DNA Concentration (ng/μl)	copies/μl	qPCR (Ct value)
10^-5^	2 × 10^-4^	0.64 × 10^5^	25
10^-6^	2 × 10^-5^	0.64 × 10^4^	29
10^-7^	2 × 10^-6^	0.64 × 10^3^	32
10^-8^	2 × 10^-7^	0.64 × 10^2^	34
10^-9^	2 × 10^-8^	0.64 × 10^1^	36
10^-10^	2 × 10^-9^	0.64	–
10^-11^	2 × 10^-10^	0.06	–

**Figure 2:**
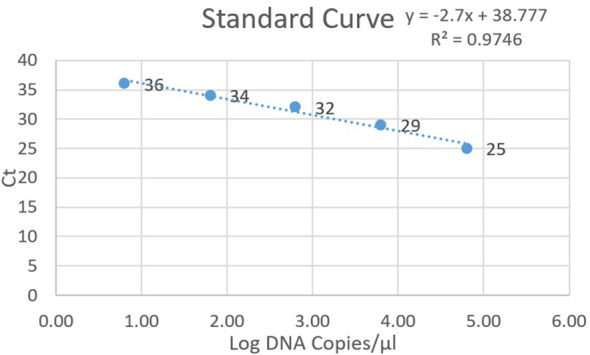
Standard curve for positive plasmid dilutions in qPCR, depicting Ct values on the y-axis and log DNA copies on the x-axis.

Values for standards and clinical specimens were computed using linear regression and documented (**Figure 2**). Nested PCR was conducted on each DNA sample using three different dilutions: the original extracted DNA, 1/10, and 1/50 dilutions, which yielded 21, 40, and 15 positives, respectively. Therefore, the most optimal result was obtained with the dilution of 1/10, indicating that 40 out of 281 samples (14%) used for setting up were positive for *P. jirovecii*. All 281 clinical samples were also subjected to detecting specific mtLSU-rRNA by qPCR, in which any samples with a Ct lower than 45 and with fluorescent peaks above 0.5 were considered positive. As a result, 40 out of 281 samples (14%) were positive for *P. jirovecii* using nested PCR. Among the positive samples, 37 out of 40 tested positive in both detection methods (nested PCR and qPCR). The remaining positive patients (n=3) who tested positive in qPCR yielded negative results in nested PCR. On the other hand, three patients had positive results in nested PCR but were negative in qPCR (**Table 3**).

**Table 3:** Comparison of the sensitivity of qPCR with nested PCR results.

		probe-based qPCR	
		positive	negative
**Nested PCR**	**positive**	37	3
	**negative**	3	238

The comparison of qPCR results with nested PCR (considered as the sensitive gold standard method) is shown in **Table 3**. The qPCR targeting the mtLSU-rRNA gene demonstrated robust diagnostic performance, with 92.5% sensitivity, 98.7% specificity, 92.5% PPV, and 98.7% NPV.

With higher PCR thresholds, specificity and NPV were improved. The lower PPV of the test was influenced by the detection of *P. jirovecii* DNA in nested PCR, suggesting that samples positive in nested PCR but negative in qPCR may indicate colonization rather than infection.

**Figure 3:**
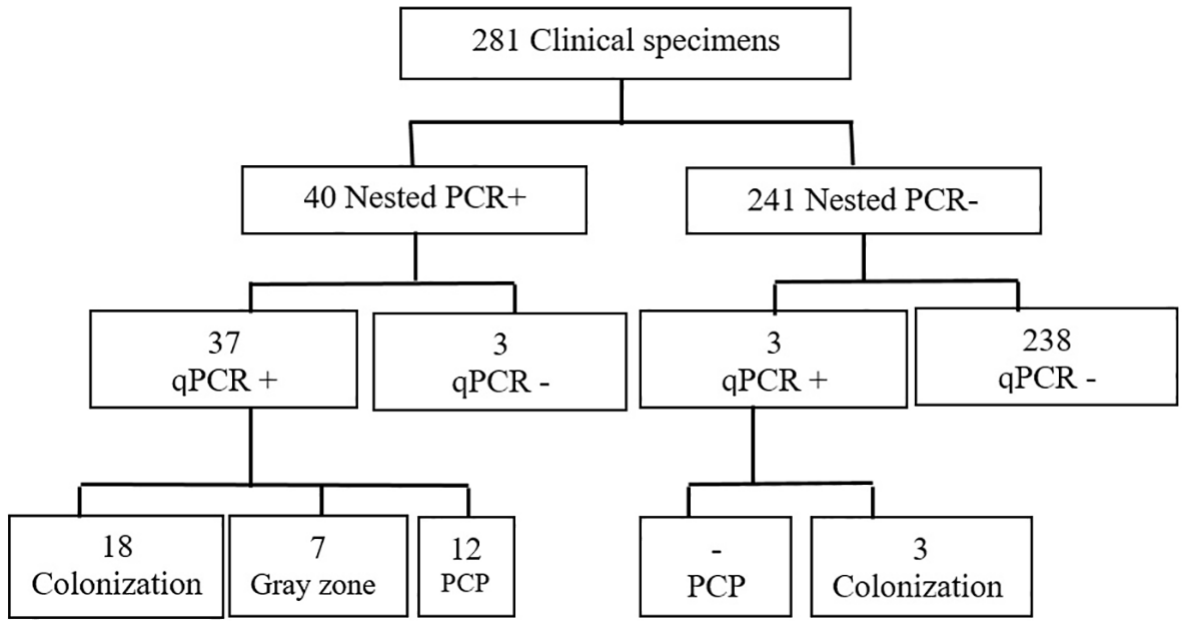
Flowchart of the study population and results.

We attempted to determine a cutoff for qPCR to facilitate the distinction between *Pneumocystis* infection and colonization and the strategic utilization of Ct and copy number quantification. Drawing on insights from prior research (Flori et al., 2004; Robert-Gangneux et al., 2014; Fauchier et al., 2016; Perret et al., 2020), we systematically refined and optimized our approach, enhancing accuracy and efficiency. To fortify the reliability of the findings, standard clone data were integrated into the clinical and experimental findings to ensure a comprehensive understanding of the dynamics associated with Ct values. Samples with Ct values >35 cycles were categorized as colonization, while those under ≤32 cycles were considered infection. With the optimal PCP cutoff, 12 patients were confirmed to have PCP infection, while 21 were identified as colonized with *Pneumocystis* (**Figure 3**). A notable grey zone remained between these Ct values (32 < x ≤ 35), introducing a dilemma for patients falling within this range. Physicians should use additional metrics to determine if a patient with these levels has PCP or is colonized. In our study, 7 patients were situated within this grey zone, exhibiting an average Ct value of 34.28. Testing of respiratory samples from the whole population of qPCR-positive patients showed a mean Ct value of 31.1 (24 to 32) for patients with PCP and a mean Ct value of 36.1 (35 to 38) for colonized patients.

40 positive *P. jirovecii* samples included 18 BAL, 12 TA, and 10 sputa. There was no discernible difference in the Ct values between the sputum, TA, and BAL samples (**Table 4**).

**Table 4:** The Ct values are different between BAL, TA, and sputum samples.

CT Value	PCP patients (n=40)			Total
	BAL	TA	Sputum	
24-26	1	–	1	2
27-29	1	2	1	4
30-32	3	3	2	8
33-35	5	4	3	12
36-38	8	3	3	14
Total	18	12	10	40

### Epidemiology results

Clinical and demographic data obtained from the information system of the hospital showed that the study population (children and adults) of the patients suspected of PCP (n=112) included 71 (63%) males and 41 (37%) females. The patients’ ages range from 0 - 93 years, with a mean of 59.7 years. All patients had one or more pulmonary disorders or other chronic diseases and had low lymphocyte counts. 32 and 80 patients were with and without COVID-19, respectively. The most common host diseases and predisposing factors were diabetes mellitus (n=21), pneumonia (n =20), heart diseases (n=12), lung nodule and abscess (n=11), trauma (n=10), COPD (n=9), asthma (n=8), surgery (n=8), lung cancers (n=7), and other cancers (n=6). 13 patients had lung damage, such as respiratory failure, influenza, tuberculosis, bronchitis, septicemia, premature, hypertension, smoking, lupus, and gastrointestinal hemorrhage. The patient’s clinical characteristics are presented in **Table 1**. The most common clinical manifestations were dyspnea (56.25%), cough (43.75%), fever (34.82%), malaise and fatigue (35.71%), chest pain (18.75%), and headache (10.71%). Pulmonary infiltrates were the most prevalent, albeit not specifically PCP-related, finding on a hest X-ray.

Considering the optimal cutoff for PCP diagnosis, two patients were confirmed to be infected with PCP, 5 were determined to be colonized with *Pneumocystis*, and one patient fell within the grey zone. The BLAST sequence analysis of four randomly chosen PCR products verified that all samples testing positive are indeed *P. jirovecii*. Analyzing the Ct values further, the mean Ct value for patients with confirmed PCP was 30 (28 to 32) with a 95% confidence interval (CI), while the mean Ct value for colonized patients was 37, ranging from 35 to 38 (95% CI). Seven patients were positive for *P. jirovecii* in both nested PCR and qPCR, but one of the specimens was positive only by probe-based qPCR. Therefore, considering the results of nested PCR and qPCR, 6.25% and 7% prevalence of *P. jirovecii* infections/colonization were rated, respectively. Among positive patients, 1.8% (2/112) had PCP with a Ct value below 32. All of these patients had underlying diseases. As shown in **Tables 1**, **5**, among patients with *P. jirovecii*, five were without COVID-19, one had just COVID-19, two were immunocompromised with COVID-19, one had COPD, and one had lung cancer. All positive samples have been recorded in the BAL. In the positive cases, the average age was 50.25 years (30–82 years), and the males (n=7) were more affected than the females. There was no significant association between the occurrence of *Pneumocystis*-positive patients and their gender or age (p>0.05), but there was a significant association between the frequency of *Pneumocystis*-positive patients and BAL samples (p<0.05). All positive patients were cured. Compared to individuals with other lung diseases, COVID-19 patients had higher rates of *Pneumocystis* colonization/infection. All patients whose PCR results were positive had some clinical signs of pneumonia caused by *P. jirovecii*; the most common symptoms were dyspnea, cough, malaise & fatigue, and fever. Most patients had image data (computerized tomography or radiological) with minor changes, with the most common ones being pulmonary nodules and opacities. Only two lung cancer patients with metastases received cotrimoxazole and had negative PCP.

**Table 5:** Features of 8 patients positive in *P. jirovecii*-specific probe-based qPCR.

Patients	Sex	Age	Type of tested sample	Underlying disease or condition	Nested-PCR result	Ct	Infection/Colonization	Outcome
1	M	30	BAL	Respiratory problems, Gastrointestinal hemorrhage	Pos	37.5	Colonization	Cured
2	M	59	BAL	Respiratory problems, Hernia surgery	Pos	34	Gray zone	Cured
3	M	45	BAL	COPD, Pneumonia, Asthma, Diabetes mellitus	Pos	37.5	Colonization	Cured
4	F	55	BAL	Respiratory problems, Heart surgery	Pos	35	Colonization	Cured
5	M	82	BAL	COVID-19, Lung cancer, Diabetes mellitus	Pos	32	Infection	Cured
6	M	44	BAL	Bronchitis, Asthma	Pos	37	Colonization	Cured
7	M	67	BAL	COVID-19	Pos	28	Infection	Cured
8	M	64	BAL	COVID-19, Trauma	Neg	38	Colonization	Cured

F, female; M, male; Neg, negative; Pos, positive.

## Discussion

PCP persists as a significant health problem among individuals afflicted with HIV and other immunocompromised patients. Without timely identification and management, PCP can become a life-threatening infection (Alshahrani et al., 2020). The lack of an *in vitro* culture method for isolating the organism is the most challenging issue in *Pneumocystis* research (Procop et al., 2004). Direct microscopy of stained smears from respiratory specimens reveals *P. jirovecii* trophic or cyst forms. Recently, nested PCR, conventional PCR, and probe/SYBRgreen qPCR have been utilized for detecting *Pneumocystis* (Arcenas et al., 2006), indicating the superiority of PCR in diagnosing PCP (Matouri et al., 2023). PCRs with mtLSU-rRNA primers have demonstrated higher sensitivity and specificity than staining techniques and serological methods (Flori et al., 2004). Multiple pieces of evidence show that *P. jirovecii* can colonize the mucosal epithelium of those with compromised immunity and healthy individuals (Linssen et al., 2006). Therefore, the role of colonization in the life cycle of *Pneumocystis* and its impact on other lung diseases is increasingly acknowledged. While nested PCR cannot differentiate between colonization and infection, qPCR, with higher speed and efficiency, less carry-over contamination, and the potential for quantification, may distinguish between infection/colonization. Our study offers new insights by addressing both methodological and epidemiological aspects of *P. jirovecii* detection. Epidemiological data were reported using both nested PCR and real time PCR.

In our study, nested PCR was considered the gold standard for developing qPCR. If all patients with detectable *P. jirovecii* DNA by the mtLSU-rRNA nested PCR technique are deemed positive, then the clinical sensitivity, specificity, and NPV of mtLSU-rRNA qPCR used in this study would be 92.5%, 98.7%, and 98.7%, respectively. Similar studies with mtLSU-rRNA qPCR found a specificity of 100% and sensitivity of 86% (Alanio et al., 2011), demonstrating comparable performances for PCP diagnosis. A high NPV of qPCR helps exclude the probability of PCP. Likewise, some commercial kits have used mtLSU-rRNA gene for diagnosis of PCP, among which BioEvolution has had a sensitivity of 73%-79.7% and a specificity of 82%-100%, and MycAssay demonstrated sensitivities of 88.9%-100% and specificities of 63.4%-92% (Guegan and Robert-Gangneux, 2019). The sensitivity and specificity displayed in our study are comparable to those of commercial kits. The assay has exhibited reliable results in effectively rolling out PCP among negative patients. However, the high sensitivity of qPCR can be deceptive, as it can detect very low fungal loads that might lead to an overdiagnosis of PCP due to misinterpretation of findings (Huggett et al., 2008; Rogina and Skvarc, 2020).

Comparing nested PCR and qPCR, 3 out of 281 samples were positive with the nested PCR but negative with the qPCR. This discrepancy could be attributed to lower sensitivity of qPCR than nested PCR. On the other hand, three samples were negative with nested PCR while positive in qPCR.

Studies have suggested adopting two qPCR cutoff values to enhance the sensitivity and specificity of the test. They suggested the presence of a grey zone with uncertain clinical significance between these two cutoff values (indeterminate zone), where distinguishing between PCP and colonization becomes challenging (Flori et al., 2004; Alanio et al., 2011; Botterel et al., 2012). Similarly, the study by Alanio et al. addressed false-negative and false-positive results by calculating a single cutoff value, resulting in 98.4% specificity and 90.5% sensitivity and consequently, 9.5% false-negative and 1.6% false-positive results for the diagnosis of PCP (Alanio et al., 2011).

The distinction between active disease and colonization in PCP detection using qPCR has been widely debated. Currently, no consensus exists regarding fungal organism load thresholds or Ct cutoffs to distinguish between disease and colonization (Belanger et al., 2023). Importantly, these cutoffs may vary across different assays or patient populations (Fauchier et al., 2016; Perret et al., 2020). For instance, immunocompromised patients without HIV often exhibit significantly lower organism concentrations compared to HIV-positive patients with PCP (Fauchier et al., 2016). In our study, aligning with previous research and incorporating plasmid cloning techniques (Flori et al., 2004; Robert-Gangneux et al., 2014; Fauchier et al., 2016; Perret et al., 2020), *P. jirovecii* detection with a Ct above the cutoff value (Ct 35) was considered colonization, while below Ct 32 as PCP. In this way, 12 (4.2%) and 21 (7.4%) samples were considered as infection and colonization, respectively. Negative qPCR results effectively ruled out the diagnosis of both colonization and PCP in 241 samples (86%). The status of seven remaining samples (2.4%), with fungal loads falling within the grey zone, was deemed undetermined. Regrettably, we lacked sufficient information to determine whether these patients were afflicted with *Pneumocystis* infection or colonization. Our study faced limitations in collecting comprehensive clinical data for these samples due to the COVID-19 pandemic.

Among the 112 patients included in the study, 7 (6.25%) tested positive with nested PCR. Nested PCR indeed had the potential to detect cases of *P. jirovecii* colonization/infection, as based on this method, we have already reported the prevalence of *Pneumocystis* in Isfahan, Iran, as 5.3% (Matouri et al., 2023). However, nested PCR might not distinguish colonization from active infection; therefore, probe-based qPCR was employed to detect *P. jirovecii*, and among the 112 analyzed patients, 8 (7%) were positive. Notably, only 1.8% (2/112) of the patients had Ct values below 32. These patients had underlying conditions, including COVID-19, lung cancer, and diabetes mellitus. Instead, 4.5% (5/112) showed Ct values above 35 and were considered as non-PCP patients (colonization cases). After thoroughly examining the clinical symptoms, the remaining samples with DNA loads falling within the grey zone classified the status as colonization. These patients displayed respiratory problems and clinical symptoms, including dyspnea, yet lacked other underlying symptoms that would warrant suspicion of *Pneumocystis* infection. Furthermore, one case was positive in probe-based qPCR but was negative in nested PCR, so all of these individuals had immunosuppressive diseases or respiratory tract symptoms, we hypothesized that *P. jirovecii* was colonizing these patients. Previous studies have reported varying rates of *P. jirovecii* colonization (20–40%) across different patient groups dependent on factors like immune status, underlying health conditions, age, and detection methods (Medrano et al., 2005; Morris and Norris, 2012). More recent research has shown that infection/colonization rates vary widely, ranging from 2.6 to 55% (Varela et al., 2003).

Considering the overlap in clinical presentation between PCP and COVID-19, applying laboratory tests and initial differential diagnosis becomes essential (White, 2021). Co-infection cases of *P. jirovecii* and COVID-19 have been reported at varying rates. Kelly et al. reported a co-infection rate as high as 22% (15 out of 69) between COVID-19 and pneumocystosis (Kelly et al., 2020), while other studies indicated rates ranging from 1.4% to 9.3% (Blaize et al., 2020; Alanio et al., 2021). In Iran, the frequency of coexistence of *Pneumocystis* with COVID-19 was reported to be 4.34% (Matouri et al., 2023). Similarly, in the present study, the rate of coexistence was 9.3% (3 out of 32 COVID-19 patients), comprising 1 case of colonization and 2 cases of infection.

It is documented that the most important variables at risk of acquiring PCP include the use of age, glucocorticoids, compromised immunity, and comorbid pulmonary diseases (Fillâtre et al., 2014). All positive patients diagnosed in our study exhibited risk factors associated with PCP development. Notably, dyspnea, cough, and fever were more prevalent in our cases than in other reports (Fujii et al., 2007; Matouri et al., 2023). The ages of our patients varied from 30 to 82, and 62.5% of them were above 50. In general, except for the BAL sample, which had a significant association with the presence of *P. jirovecii* (p<0.05), there was no significant relationship between gender, age, and clinical symptoms with the presence of this microorganism (p>0.05).

## Conclusion

In this study, the probe-based qPCR targeting mtLSU-rRNA gene exhibited good diagnostic performance with a sensitivity of 92.5% and specificity of 98.7%. The overall *Pneumocystis* infection/colonization detection rate in the Kashan samples was 7% (8/112). Ct values were suggested to differentiate colonization from pneumonia in immunocompromised patients. When Ct values fall within the gray zone, physicians must decide between prophylactic or curative treatments based on other clinical/paraclinical findings. To further augment the diagnostic sensitivity, it is highly recommended to integrate qPCR results with clinical parameters and biomarkers, promising to offer a more precise and comprehensive understanding of *Pneumocystis*-related conditions.

## Data Availability

The original contributions presented in the study are included in the article/supplementary materials. Further inquiries can be directed to the corresponding author.
